# Prophylactic Human Papillomavirus Vaccination: From the Origin to the Current State

**DOI:** 10.3390/vaccines10111912

**Published:** 2022-11-11

**Authors:** Ayazhan Akhatova, Azliyati Azizan, Kuralay Atageldiyeva, Aiymkul Ashimkhanova, Aizada Marat, Yerbolat Iztleuov, Assem Suleimenova, Saikal Shamkeeva, Gulzhanat Aimagambetova

**Affiliations:** 1 School of Medicine, Nazarbayev University, Astana 010000, Kazakhstan; 2 Department of Basic Sciences, College of Osteopathic Medicine, Touro University, Henderson, NV 89014, USA; 3 Department of Medicine, School of Medicine, Nazarbayev University, Astana 010000, Kazakhstan; 4 Clinical Academic Department of Internal Medicine, CF University Medical Center, Astana 10000, Kazakhstan; 5 Department of Obstetrics and Gynecology #1, NJSC “Astana Medical University”, Astana 010000, Kazakhstan; 6 Medical Center, Marat Ospanov West-Kazakhstan Medical University, Aktobe 030000, Kazakhstan; 7 Kazakh Institute of Oncology and Radiology, Almaty 050000, Kazakhstan; 8 Institute of Laboratory Medicine, Clinical Chemistry and Molecular Diagnostics, Leipzig University Hospital, 04103 Leipzig, Germany; 9 Department of Biomedical Sciences, School of Medicine, Nazarbayev University, Astana 010000, Kazakhstan

**Keywords:** HPV, prophylactic HPV vaccine, HPV vaccination, HPV vaccination acceptance, cervical cancer prevention, Gardasil, Cecolin, Gardasil-9, Cervarix

## Abstract

Immunization is the most successful method in preventing and controlling infectious diseases, which has helped saving millions of lives worldwide. The discovery of the human papillomavirus (HPV) infection being associated with a variety of benign conditions and cancers has driven the development of prophylactic HPV vaccines. Currently, four HPV vaccines are available on the pharmaceutical market: Cervarix, Gardasil, Gardasil-9, and the recently developed Cecolin. Multiple studies have proven the HPV vaccines’ safety and efficacy in preventing HPV-related diseases. Since 2006, when the first HPV vaccine was approved, more than 100 World Health Organization member countries reported the implementation of HPV immunization. However, HPV vaccination dread, concerns about its safety, and associated adverse outcomes have a significant impact on the HPV vaccine implementation campaigns all over the world. Many developed countries have successfully implemented HPV immunization and achieved tremendous progress in preventing HPV-related conditions. However, there are still many countries worldwide which have not created, or have not yet implemented, HPV vaccination campaigns, or have failed due to deficient realization plans associated with establishing successful HPV vaccination programs. Lack of proper HPV information campaigns, negative media reflection, and numerous myths and fake information have led to HPV vaccine rejection in many states. Thus, context-specific health educational interventions on HPV vaccination safety, effectiveness, and benefits are important to increase the vaccines’ acceptance for efficacious prevention of HPV-associated conditions.

## 1. Introduction

Vaccines are one of the greatest healthcare successes of the 20th century, which facilitated a significant reduction in morbidity and mortality from diverse infectious agents [1,2,3].

The discovery of the causative role of human papillomavirus (HPV) in the development and progression of various benign and malignant conditions is considered one of the most significant events in medicine and global healthcare [4,5,6]. It initiated the cascade of medical novelties, which had a tremendous impact both on the health conditions of individual lives and the sociocultural situation of many countries [7,8,9,10].

According to the Centers for Disease Control (CDC), HPV is linked to more than 90% of anal cancer and cervical cancer cases, nearly 70% of vaginal cancer cases, and more than 60% of penile malignancy cases [11]. These diseases were considered to be unpreventable before the era of HPV vaccination. The discovery of the HPV infection association with various benign and malignant conditions and the development of preventive HPV vaccines have led to revolutionary changes in the prevention of the abovementioned conditions [12]. The prophylactic HPV vaccines are effective at preventing HPV infection and HPV-associated neoplastic conditions. However, these vaccines provide no therapeutic benefit and cannot eliminate pre-existing infections [13,14].

The worldwide HPV vaccination coverage in 2018 was reported to be 12.2% [12]. Currently, around 120 million women have received at least one dose of a prophylactic HPV vaccine globally [15,16]. According to calculations in 2019, about 15% of girls and 4% of boys were vaccinated with the full course of the HPV vaccine, and 20% of girls and 5% of boys received at least a single dose [17]. However, millions of girls worldwide, mostly in low- and middle-income countries (LMICs), are left without the HPV vaccination, and over 300,000 women died of cervical cancer in 2018, 90% of whom were in LMICs [12]. Out of 61 million girls who reached the age of 15 years in 2018, 7000 may develop cervical cancer and die from this disease in their lifetime [17].

## 2. Harald zur Hausen’s Investigations on HPV

**Figure 1:**
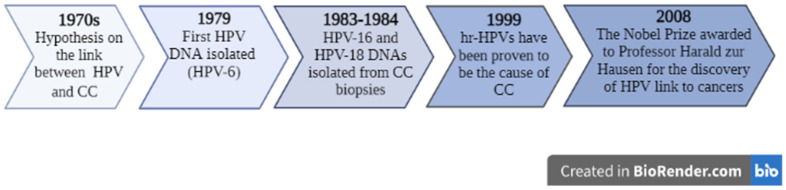
Establishing the link between HPV and cancers. Figure legend: HPV—human papillomavirus; CC—cervical cancer.

Very early investigations of cervical cancer etiologies are now known to stem from the 19th-century works of Rigoni-Stern, who established a greater prevalence of cervical cancer among sexually active women compared to virgins and nuns [18,19,20]. Later in the 1960s, scientists’ attempts to link cervical cancer to herpes simplex virus (HSV) type 2 failed after a large prospective study [21]. In the 1970s, the German scientist, Harald zur Hausen, continued searching for an infectious agent linked to cervical cancer (Figure 1) [5,22]. In the 1980s, acknowledgment of the HPV infection’s contribution to cervical cancer development reinforced the need to study the HPV-associated cell proliferation pathogenesis [6].

Tremendous changes in the understanding of cervical cancer etiology and natural history have appeared after zur Hausen’s suggestion that cervical cancer could have an infectious etiology [6,21,23]. Considering his unsuccessful attempts to extract HSV type 2 from cervical cancer biopsies and the fact that genital warts were caused by papillomavirus particles, the focus of his subsequent studies shifted toward the genital warts virus [21,23]. After studying large numbers of wart biopsies, in 1974 he published his report demonstrating a cross-hybridization of plantar warts’ virus DNA with some other warts, except for genital warts and cervical cancer biopsy samples [24,25]. It was the first sign of the existence of different types of papillomaviruses.

In 1979, Lutz Gissmann and Ethel-Michele de Villiers isolated the first DNA of HPV-6 from genital warts, which was not isolated from cervical cancer biopsies [26,27,28,29]. However, the HPV-6 DNA probe was useful to isolate the DNA of HPV-11 from laryngeal papilloma [26,30]. The HPV-11 probe was later identified in the tissue of cervical cancer biopsies [26,31]. There were also unclear bands of HPV-11 DNA in some probes, which suggested the presence of other related HPV types [26]. zur Hausen’s successors, Mathias Dürst and Michael Boshart, contributed to his work by cloning these bands [23,26]. Eventually, in 1983 they managed to isolate HPV-16 and in 1984 they isolated HPV-18 DNA (Figure 1) [23,26]. Their results showed the presence of HPV-16 DNA in approximately 50% of cervical cancer biopsy samples, whereas HPV-18 DNA was present in about 20% of different cervical cancer cell lines. The role of HPV-16 and HPV-18 became more apparent as they continued their work, as they identified them being integrated into the host’s genome [5]. Moreover, certain parts of viral DNA systematically became lost during the integration process, whereas some genes, later designated as E6 and E7, were continuously transcribed in cancer cells [5,21]. Harald zur Hausen found that precursor lesions contained these viral particles and genes as well. However, his attempts to connect with pharmaceutical companies for the development of preventative HPV vaccines were unsuccessful due to the unavailability of the market. Nevertheless, his contribution to an “understanding of the mechanism of HPV-mediated carcinogenesis” was finally and totally acknowledged by the scientific society, and Professor Harald zur Hausen was awarded one-half of the Nobel Prize in Medicine or Physiology in 2008 for his investigations on the HPV link to cancers [19,26,32].

## 3. Prophylactic HPV Vaccine Development

In 1981, Professor Ian Frazer began his research career at the University of Queensland working on hepatitis B and the causes of liver diseases [6,19,20]. After meeting a liver disease expert in Germany, who happened to be a student of Professor zur Hausen, Professor Frazer was convinced by his colleague to change the course of his research to closely look at a new virus which causes cancer [19,33]. As he simultaneously worked with HIV-positive patients, it was evident that genital warts caused by HPV also posed a problem for these patients [19], and he later found out that HPV caused not only cancer of the cervix, but of the anal passage as well [19,34]. The immunology of HPV was unknown at the time and neither serological tests for HPV nor in vitro systems to study the viral propagation were available [19,35].

In order to elucidate the mechanism of cancerogenesis and to study the effect of persistent infection of the skin by HPV, Professor Frazer visited Dr. Margaret Stanley’s laboratory at Cambridge to work on mouse transgenic models of the HPV E6 and E7 oncoproteins [19,36]. Although the experiment failed, this visit led to a historical meeting with Dr. Jian Zhou, a virologist from China, who was interested in HPV as well [6,19].

**Figure 2:**
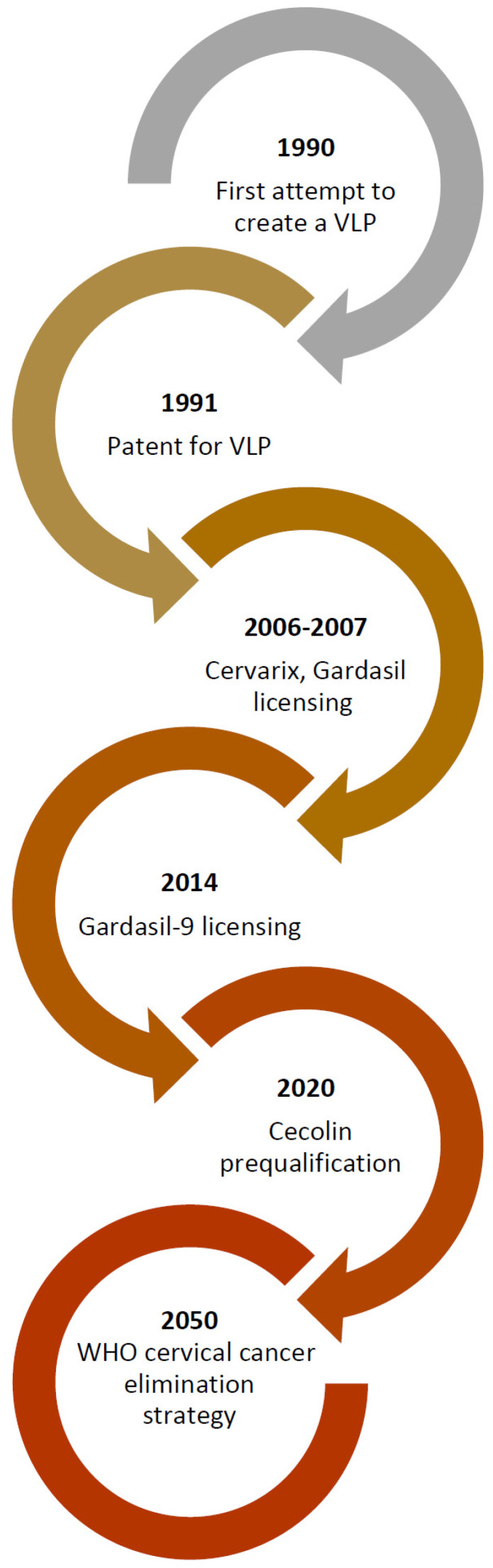
HPV vaccine development. Figure legend: VLP—virus-like particle; WHO—World Health Organization.

The ideas of two genius minds resulted in a series of experiments. In 1990, they started working together to build the papillomavirus shell containing viral capsid proteins in monkey kidney cells using a recombinant vaccinia virus vector (Figure 2) [19,37,38,39,40]. The attempts to assemble a viral capsid consisting of 360 copies of L1 and a diverse number of L2 proteins from the gene clones provided by the Gissmann laboratory took almost a year. This effort led them to successfully build something resembling the viral capsid [39]. The important lesson that the researchers learned while creating this construct is the necessity of obtaining the L1 major capsid gene expression from the second translation initiation codon, as well as changing the Vaccinia transcriptional promoter sequence and co-expressing the L2 [19,39]. Thus, in 1991 they filed a patent for the creation this construct, known as viral-like particles (VLPs), as they believed it could be a key for the future vaccine development [19,39]. It was the starting point of the production process of a functional vaccine, the resolution of which Dr. Zhou could not witness himself, as he passed away in 1999 [19,41].

There were multiple challenges on the way to success in the production of the first HPV vaccine as business developers and vaccine production companies were not convinced at the beginning about the necessity and the convenience of the market for such a vaccine [6,19,33].

In order to persuade the vaccine companies such as Merck and GlaxoSmithKline (GSK) of the importance of developing HPV preventive vaccine based on VLPs, it was necessary to prove that high-risk HPVs linked to cervical cancer are rather common among the female population [19,33]. Studies among college students have shown that at least 50% of sexually active women, specifically young women, could be infected with high-risk HPV types within 3 years of their college lives [19,42]. Although the vast majority have complete viral clearance, some people would still develop precancerous lesions [19]. In 2006, Merck started marketing a quadrivalent HPV vaccine under the name Gardasil, which was followed by the vaccine Cervarix, produced using a similar technique in 2007 by the British company GSK [43]. Over time, the vaccine formulations and indications have only been improving to amplify its efficacy. The FUTURE clinical trial, phases I, II, and III, which were performed among 5455 and 12,167 women in 16 countries, have shown a higher than 90% efficacy in the prevention of precancerous lesions [10]. The triumph of this vaccine is undoubted: it is now used in about 120 countries, saving 300,000 people each year from this fatal disease [43,44,45,46,47].

## 4. Prophylactic HPV Vaccines’ Mechanism of Action

Prophylactic HPV vaccines’ mechanism of action is based on a deep understanding of HPV infection pathogenesis. Thus, in order to understand the mechanism of action of HPV vaccines, the pathogenesis of HPV infection needs to be revisited and fully elucidated.

HPV is a double-stranded circular DNA virus with a genome consisting of nearly 8000 base pairs [15,48]. After initial infection, the clinically apparent HPV infection is cleared out by the host’s natural immunity within 12–24 months [15,49,50,51,52] and does not develop into cervical cancer. However, in 10% of cases, patients suffer from persistent infection, which is essential for the development of dysplastic lesions [49,50]. The persisting HPV infection enters the basal epithelial cells through epithelial breaches in areas that are particularly sensitive to malignant transformations [15,50,51,52]. The genome of HPV consists of early (E1, E2, E4, E5, E6, and E7) and late genes (L1 and L2), which are assembled into progeny viral particles in host cells [48,50,51]. The most important HPV genes in terms of their cancerogenic properties are the E6 and E7 oncoproteins/genes [48,51]. The longer the infection persists and the more the viral life cycle is repeated, the greater the risk for cervical intraepithelial neoplasia (CIN) of grades I, II, and III, or invasive carcinoma development [50,51,52].

In order to clear the HPV infection, the host immune system activates CD4+ and CD8+ lymphocytes, macrophages, and natural killer cells, and produces neutralizing antibodies (NAbs). These HPV-specific NAbs target only viral particles, leaving infected cells out [49,50,51,52]. The effector T-cells recognize early viral proteins and eliminate virus-infected cells [15]. The helper T-cells recognize the viral L1 protein and induce a neutralizing antibody response, which prevents viral dissemination and reinfection of the host [15]. If, at this point, immune cells fail to clear the virus, the oncoproteins E6 and E7 induce lesion progression as the result of methylation of the E2 promoter and integration of the viral DNA [15,50,51,52]. The E6 oncoprotein interferes with the function of apoptosis regulator protein p53 [48,51,53], which leads to promoting cell survival and extended cell life [15,51]. The E7 is a small oncoprotein with 127 amino acids, which is able to bind to hosts’ retinoblastoma protein (pRb), which physiologically functions as a tumor suppressor [48]. The HPV E7 oncoprotein binding to pRb results in pRb phosphorylation, and subsequent cells’ continuous entry into the S-phase gives rise to uncontrolled cell proliferation [15,48,51]. Thus, the E6 and E7 oncoproteins act synergistically, causing cell mutations, genomic instability, and loss of apoptosis, which forces a cell repetitive proliferation [15,48,51,52].

The main purpose of preventative HPV vaccines is to induce a neutralizing antibody response to viral particles [15,20]; hence, the prevention of subsequent HPV infection. Since cervical tissue lacks secondary lymphoid tissues, where memory B-cells are stored, the immunity acquired from natural infection is insufficient to protect from repeated infections [15,50]. Moreover, in order to protect a sexually active person from an HPV persistent infection, the levels of NAbs should remain high and sustained. HPV vaccines contain VLPs from recombinant HPV capsid proteins that lack viral DNA, and are thus lacking infectious and carcinogenic capacities [20,48,50]. The recombinant L1 protein self-assembles into VLPs, mimicking a native virion due to the morphological resemblance [50]. As these particles are recognized by host antigen-presenting cells (APCs), the cascade of immune responses induces the formation of different type-specific NAbs, which bind to the viral particles and prevent their uptake by the host target cells [20,50]. Thus, the L1-VLP-based vaccines elicit NAbs, which are produced against conformational epitopes within the surface loops of the capsid [54].

According to recent reports, the cellular immune response has been known to be crucial in the regression of the cervical lesions. This is due to the increased CD4:CD8 lymphocyte ratio in the cervical stroma, the CD4+ T-cell helper responses to E2, and the high amount of CD8+ T-cells against E6 and E7 proteins in spontaneously regressed tissues [14,50].

## 5. Prophylactic HPV Vaccines: Types, Safety, and Immunogenicity

### 5.1. Types

Currently, there are four prophylactic HPV vaccines available [20,55,56,57,58,59,60,61,62,63,64,65,66,67,68,69,70,71,72,73]: (1) a bivalent HPV vaccine targeting HPV-16 and HPV-18 (Cervarix, GSK, Rixensart, Belgium); (2) a quadrivalent HPV vaccine targeting HPV-6, HPV-11, HPV-16, and HPV-18 (Gardasil, Merck, Sharp & Dohme (Merck & Co., Whitehouse Station, NJ, USA)) [71,72,73]; (3) a nonavalent HPV (9vHPV) vaccine targeting HPV-6, HPV-11, HPV-16, HPV-18, HPV-31, HPV-33, HPV-45, HPV-52, and HPV-58 (Gardasil-9, Merck, Sharp & Dohme (Merck & Co., Whitehouse Station, NJ, USA)) [7,10,15,54,56,60]; and (4) a bivalent HPV vaccine that targets HPV-16 and HPV-18 (Cecolin, Xiamen Innovax Biotech Co., Ltd., Xiamen, China), (Table 1) [20,54,55,57,58,59,70].

The first vaccines were approved in 2006 and 2007 [7,19], and within 10 years, were implemented in more than 80 countries worldwide [54]. Gardasil-9 was approved in 2014 [7]. Cecolin was approved by the Chinese National Medical Products Administration in December 2019 and officially initiated in China (excluding Hong Kong, Macao, and Taiwan) in May 2020 [58]. This new vaccine was prequalified by the World Health Organization (WHO) in October 2021 [57,59].

All prophylactic vaccines contain synthetically manufactured VLPs of the L1 epitope [7]. In comparison with the bivalent vaccines, a quadrivalent vaccine additionally includes L1-VLPs of HPV-6 and HPV-11, which are common causes of skin papillomas [54], thus protecting from genital warts. The nonavalent vaccine, if compared with the bivalent, has increased concentrations of L1-VLPs for HPV-16 and HPV-18; if compared to the quadrivalent, it has L1-VLPs of five additional types (HPV-31, HPV-34, HPV-33, HPV-52, and HPV-58) [7,54,60].

However, since all currently available HPV vaccines are based on L1-VLPs, they are able to induce mostly type-specific immune responses [54]. This is one limitation of these vaccines, although some studies suggest a minor ability of L1-VLP-based vaccines to elicit a cross-protection against phylogenetically related types [54,61].

#### 5.1.1. Bivalent Prophylactic HPV Vaccines: Cervarix and Cecolin

There are two bivalent vaccines developed to prevent HPV-16 and HPV-18 infection: Cervarix by GSK and Cecolin by Xiamen Innovax Biotech Co., Ltd.

##### Cervarix

The bivalent HPV prophylactic vaccine Cervarix was the first ever available HPV vaccine, produced by GSK and approved in 2007 for the prevention of HPV-related cervical cancer [10,56]. Cervarix has been approved for use in females 9–25 years of age (Table 1) [62]. The baculovirus expression system was used to produce the recombinant form of the L1-VLP HPV antigen, which was coupled to the AS04 adjuvant made of 500 μg of aluminum hydroxide and 50 μg of 3-O-desacyl-4′-monophosphoryl lipid A [10,54,62]. Cervarix contains 20 μg each of HPV-16 and HPV-18 L1-VLPs. A series of three intramuscular 0.5 mL injections at months 0, 1, and 6 are recommended for girls aged 11 to 13 for this vaccine [10]. The efficacy of Cervarix for the prevention of persistent HPV-16 infection in previously uninfected adult women was reported to be at the level of 83% (95% CI = 71% to 90%), and for the HPV-18 type, at the level of 43% (95% CI = 31% to 53%) [56,63]. Moreover, according to Schauner and Lyon (2010), Cervarix has been proven to be 93% effective in the prevention of CIN (grade I-II) and adenocarcinoma in situ (AIS) in a follow-up period of 14 to 44 months for patients with previous or ongoing HPV infection [54,64]. According to other reports, the bivalent vaccine efficacy against CIN II-III associated with HPV-16 and HPV-18 was 70% (95% CI = 19% to 89%) [56,63]. Thus, the vaccine has been proven to have high efficacy against conditions linked with HPV-16 and HPV-18 types [56,65].

Compared to Gardasil, Cervarix has not been shown to be effective in the prophylaxis of cervical cancer [64]. However, the results of a single head-to-head study showed that the amount of antibodies produced against HPV-16 and HPV-18 after administration of Cervarix at 1 month was several-fold higher when compared to Gardasil [64]. The association with a higher efficacy in the prevention of cervical dysplasia or cancer of such in-creased immunogenicity is yet unknown. The immune protection against HPV-16 and HPV-18 lasts for as long as 6 years, but whether a booster dose is required remains to be seen [64,66].

However, there are many other studies concerning the persistence of antibody responses following HPV vaccination [66,67]. Some of them predicted that the mean of bivalent vaccine-induced HPV-16 and HPV-18 antibody levels will remain high and above those associated with natural infection for a minimum of 20 years [67]. However, the vaccine investigator and inventor, Ian Fraser, projected the duration of postvaccination HPV-16 antibodies to range from 12 years to nearly life-long in the majority of HPV vaccines [66,68]. However, more studies on yearly antibody levels and the potential protection of vaccinated individuals would help to improve our understanding.

##### Cecolin

The bivalent HPV vaccine Cecolin is a mixture of two aluminum hydroxide adjuvant-adsorbed recombinant L1 capsid proteins of HPV-16 and HPV-18, each self-assembled into VLPs. The formulation comprises 40 μg of HPV-16 and 20 μg of HPV-18 L1-VLPs suspended in 0.5 mL of buffered saline containing 208 μg of aluminum adjuvant [2,58,69]. The HPV-16 and HPV-18 L1 antigens are expressed in Escherichia coli by the recombinant DNA technology [58,69]. Cecolin is an injectable vaccine and should be administered intramuscularly.

Cecolin has been available in China since 2020 [57] and is indicated for women aged 9–45 years (Table 1) [58,69]. The vaccine is recommended as a preventative measure for CIN I-III, AIS, and persistent infection with HPV-16 and HPV-18. At present, the vaccine has not demonstrated a preventative effect on individuals who have been already infected by HPV-16 and HPV-18 [58,69]. The three-dose immunization was originally approved with a flexible regimen: the second dose of Cecolin can be injected within 1–2 months after the first dose, and the third dose can be injected within 5–8 months after the first dose [58,69]. However, since Cecolin’s two-dose regimen immunogenicity has not been shown to be superior to a three-dose schedule in young women, a two-dose schedule is approved for girls aged 9–14 years, in addition to the original three-dose regimen [70]. Currently, there is no available data that could dictate whether a booster vaccination is required.

The advantage of Cecolin [57] is in its price; the three-dose cost is less than the three-dose cost of Gardasil and Cervarix [70]. However, cost-effectiveness should be evaluated against the gross domestic product (GDP) per capita in particular countries [57].

#### 5.1.2. Quadrivalent Prophylactic HPV Vaccine: Gardasil

The quadrivalent HPV vaccine Gardasil was licensed in 2006 for the prevention of genital warts; cervical, vulvovaginal, anal intraepithelial lesions; and cancer in women and men aged 9–26 years (Table 1) [71,72,73]. Today, the quadrivalent vaccine is available in 129 counties to prevent persistent infection with HPV-6, -11, -16, and -18, which are associated with low-grade and high-grade CIN, AIS, cervical cancer, high-grade vaginal and vulvar intraepithelial neoplasia (VIN), vulvar cancer, high-grade anal intraepithelial neoplasia (AIN), anal cancer, and anogenital warts (Table 1) [71,72]. The Gardasil vaccine is produced using the S. cerevisiae expression system, and aluminum hydroxyphosphate sulfate is used as an adjuvant [10]. Gardasil is composed of VLPs expressing the L1 capsid protein [74]. The composition of a 0.5 mL dose of the quadrivalent HPV vaccine is as follows: 20 μg of the HPV-6 L1 protein, 40 μg of HPV-11, 40 μg of HPV-16, and 20 μg of the HPV-18 L1 protein [72,73,74].

This vaccine is also recommended for girls and boys 11–12 years old, whilst there is a possibility to obtain a concurrent catch-up vaccination for the older generation. The original three-dose regimen at 0, 2, and 6 months was recently suggested by the WHO to be changed to a two-dose regimen 6 or 12 months apart, as the immunogenicity with two doses in prepubertal girls was noninferior to the immunogenicity with three doses in women aged 16–26 [75]. The vaccine protection lasts for up to 8 years [72,73,74].

The prophylactic efficacy has been evaluated in a number of phase II and III randomized, double-blind, placebo-controlled, multicenter studies, such as FUTURE I, II, and III, among more than 30,000 women of different age categories [74]. The studies evaluated samples taken from Pap smears and HPV DNA assays, as well as anti-HPV serum antibodies on day 1 and after particular time intervals from more than 29,000 men and women [74]. The results of FUTURE I comparing the efficacy of the quadrivalent vaccine to the placebo was 100% for the prevention of external anogenital, vaginal, and cervical lesions after a follow-up of 3 years in women aged 15–26 [74]. It was most efficient against HPV-16 and -18 (95%), whereas for HPV-6 and -11, efficacy was 75% [74]. The FUTURE III study involving older women aged 24–45 and was 91% efficient in the prevention of persistent infection and cervical and external genital diseases associated with HPV-6, -11, -16, and -18, as well as being 83% efficient only against HPV-16 and -18 types at a follow-up of 2.2 years [72,74]. Among 684 patients followed for 6.26 years, there were no new cases of HPV-related CIN or external genitalia lesions [74]. Regarding boys/men, the V501-020 trial compared the vaccine efficacy with a placebo in preventing external genital lesions among the 16–26-year-old population, which resulted in a 66% efficacy in the “intent-to-treat” population and 90% in the “per-protocol susceptible” population [74].

#### 5.1.3. Nonavalent Prophylactic HPV Vaccine: Gardasil-9

The nonavalent HPV vaccine Gardasil-9 was licensed in 2014 for the prevention of genital warts; cervical, vulvovaginal, anal intraepithelial lesions; and cancer in women and men aged 9–45 years, covering five additional HPV strains compared to Gardasil (HPV-31, -33, -45, -52, and -58) [76,77]. It has the same vaccine formulation as the quadrivalent vaccine, except for 500 μg of AAHS instead of 225 μg [60,76]. Gardasil-9 is indicated for boys and men 9–45 years of age for the prevention of anal, oropharyngeal, and other head and neck cancers caused by HPV-16, -18, -31, -33, -45, -52, and -58 (Table 1) [60].

In phase III trials and clinical studies, the efficacy of the quadrivalent and nonavalent HPV vaccines for the prevention of HPV-associated CIN I and II, and vulvar and vaginal intraepithelial lesions grade 2 and 3, was compared among 14,000 females aged 16–26 [56,76]. The efficacy of the nonavalent vaccine in the per-protocol population for the prevention of the previously mentioned conditions was 96.7%, whereas the efficacy for the prevention of only CIN I and III associated with HPV-31, -33, -45, -52, and -58 comprised 96.3% and 96.0% for a 6-month persistent infection with more than a 99% seroconversion to all nine HPV types [56,76]. The trial investigated the immunogenicity of the Gardasil-9 vaccine among 2400 women and men aged 9–16 years in comparison with 400 females aged 16–26 [76].

A German study to evaluate the public health effects and cost-effectiveness of vaccination with Gardasil-9 indicated that the vaccination of boys with the nonavalent vaccine decreased the incidence of cervical cancer by 24% [78]. This study also reported a reduction in anal cancers in men by 30% and in women by 14% [78].

### 5.2. Safety

Multiple reports have been published on the HPV vaccines’ local and general side effects, investigating and reporting on the vaccines’ safety [56,79,80,81,82].

A systematic review that analyzed a total of 29,540 individuals reported that the most frequent adverse reaction associated with the HPV vaccines was pain and edema at the injection site, followed by fever, fatigue, and headaches [1,83].

In the HPV vaccines safety report by Arbyn et al. (2018), the risk of serious adverse events was shown to be similar in control and HPV-vaccinated women of all ages [79]. In the same study, the mortality rate was not found to be significantly different in control and HPV-vaccinated individuals [79]. Moreover, the study results did not reveal an increased risk of miscarriage among those who became pregnant during the investigation [79]. The effects on the rates of fetal congenital abnormalities and stillbirths were reported as uncertain.

Based on the available data, local symptoms were reported as the most common side effect [56,74]. However, a comparative study on the immunogenicity and safety between the bivalent vaccine Cervarix and the quadrivalent vaccine Gardasil suggested a higher incidence of injection-site reactions and other symptoms after immunization with Cervarix [2,84]. Overall, no increased risk of serious adverse effects was observed [2,56,79,84]. However, some researchers reported cases of immune system activation after HPV vaccination [85,86]. Further studies should aim to identify a genetic background predisposed to HPV vaccine-related adverse reactions [85].

### 5.3. Immunogenicity

Immunogenicity is the capacity to create an immune response that could be measured either by antibody formation or cell-mediated immunity via cytotoxic T-lymphocytes [87].

In the case of HPV vaccine immunogenicity investigations, the majority of studies measure serum-neutralizing antibodies as an immune response correlate [87,88,89]. However, cell-mediated immune responses may provide protection against the progression of HPV infection to CIN III or further [89].

Bivalent and quadrivalent HPV vaccines induce excellent protection against persistent HPV-16 and HPV-18 types [63,89,90]. Numerous studies have shown a significant seroconversion to all targeted HPV types in women 15–26 years of age (99–100%) [15,20,56,63,76,88,89]. Moreover, the immune response to the HPV vaccines is higher than from natural infection, where seroconversion is reported in 50–70% of HPV-infected females and 2–51% of males [56,91].

A comparison of the immunogenicity between the bivalent vaccine Cervarix and quadrivalent vaccine Gardasil in healthy adult women aged 18–45 years showed serum-neutralizing antibody responses for HPV-16 and HPV-18 types induced by the bivalent vaccine to be 7.8-fold (in the 18–26-year-old group), 5.6-fold (in the 27–35-year-old group), and 2.3-fold (in the 36–45-year-old group) higher than the levels observed with the quadrivalent vaccine (*p* < 0.0001) [56,92]. According to another comparative study, the nonavalent and quadrivalent vaccines offer similar protection against cervical, vaginal, and vulvar precancerous lesions [93].

However, the age-related difference in immune response was documented with vaccine-induced antibody levels being higher in teenage girls and boys than in young women [2,56,72,79]. Even though levels of antibody titers may be indicative of protection against HPV-16 and HPV-18 for women until their sixties, and potentially, of lifelong immunity, immunization with HPV vaccines should be received in the early teens [2,79]. A subsequent booster dose may not be required.

The study on the immunogenicity of the bivalent vaccine Cecolin involving women aged 9–26 years (NCT02562508) [94] showed noninferiority for levels of IgG in 9–17-year-old girls receiving three doses compared with adults [94]. Noninferiority was also demonstrated for NAbs in the same study. However, more studies are required to investigate Cecolin’s immunogenicity as the vaccine is new and its characteristics are yet to be evaluated.

Although the HPV vaccine efficacy is doubtless [20,63,89], further investigations to identify a definitive immune correlate, other than serum-neutralizing antibodies, for the HPV vaccination would be valuable [87].

## 6. Vaccine Dosing Regimens

### 6.1. Three-Dose Schedule

Vaccine schedules are usually planned with the aim of producing a strong and sustained antibody response for possible future infections. A three-dose regimen is a typical regimen for vaccines based on inactivated proteins for infants [93]. The second dose is given at an interval of one or two months after the first dose; the third dose is given six months after the first dose [93]. The first two doses induce the production of immune memory B-cells in the bone marrow, and thus, are called “prime doses”. The second dose generates a greater antibody response than the first dose, with increased affinity for the antigen [93]. As a result of this process of “affinity maturation”, B-lymphocytes with high affinity are produced and differentiated into B-memory cells that are able to quickly respond to an antigen and produce NAbs [93]. In the study by Schiller and Lowy (2018), it is suggested that multiple repeated doses are not required for affinity maturation [95]. Due to the morphological resemblance of VLPs to an actual infection, the continuous production of long-lasting plasma cells ensures a strong, sustained immune response with reduced doses [93].

Conflicting data exist on the efficacy of the two-dose schedule over the three-dose schedule vaccination. In a secondary analysis of data from a randomized clinical trial (RCT) conducted more than a decade ago, Kreimer et al. (2011) observed that fewer doses received by 20% of the study population (women aged 18–25 years) in Costa Rica showed a similar incidence of HPV infection that persisted for >12 months compared to the population with a three-dose schedule [20,96,97]. According to the study, of the total vaccinated cohort, the vaccine efficacy against HPV-16 and HPV-18 incident infection that persisted for ≥12 months was 83.7% with two doses, and 92.6% with three doses [96].

The Indian RCT among girls aged 10–18 years vaccinated with the quadrivalent vaccine Gardasil has also demonstrated that participants who received three doses had an incidence rate of infection of 0.4% (95% CI—0.0 to 1.3%, 2/536), whereas the two-dose schedule was associated with a higher incidence of 0.8% (95% CI—0.2 to 1.9%, 4/526) [98], but this study did not take into account the poorer immunogenicity when the interval between two doses is 1 month rather than 6 months or more.

Nowadays, the data demonstrate that fewer than three doses of Cervarix and Gardasil provide protection against HPV infection and associated conditions [20,90,99]. In the Costa Rica vaccine trial (CVT), one, two, and three doses of the bivalent HPV vaccine resulted in equal protection against incident, 1-year or longer persistent HPV-16 and HPV-18 infection over the 4-year period of the trial [99,100].

In a Swedish study, one million women aged 10–24 years were followed-up for 6 months [101]. The study found that the risk of genital warts decreased by almost 40% with each dose of HPV vaccination (0 vs. 1, 1 vs. 2, and 2 vs. 3), with an overall reduction of 80% by three doses of the quadrivalent vaccine when compared with controls who were not vaccinated [101].

### 6.2. Two-Dose Schedule

Currently, available data demonstrate that fewer than three doses of bivalent and quadrivalent vaccines are at least partially prophylactic for preventing HPV-associated conditions [72]. The significance of reducing the dosage is shown in the strategy to increase coverage by simplifying the immunization schedule as much as possible [102]. After the WHO approved a two-dose schedule for HPV immunization in 2014, 80 countries have fully implemented HPV immunization; 4 countries have it partially implemented; and 65 countries have introduced the two-dose schedule into their national immunization scale among 9–14 years old girls [93].

In the study by Iversen et al. (2016), for Gardasil-9, a two-dose schedule (0 and 6 or 0 and 12 months) in girls and boys aged 9–14 years, and a three-dose schedule (0, 2, and 6 months) in women aged 16–26 years, were compared [20,103]. In this study, the authors found that among girls and boys receiving two-dose regimens of Gardasil-9, separated by 6 or 12 months, immunogenicity 4 weeks after the last dose was noninferior to a three-dose schedule.

According to a Cochrane review of 31,940 immunized participants with follow-ups up to 5 years, the immunogenicity of the two-dose HPV vaccine was similar to the three-dose schedule. Antibody response was stronger with a longer interval of 6 or 12 months between the first and the second doses than with an interval of fewer than 6 months [93].

The analysis of data from the CVT and PATRICIA trial, which aimed to assess the efficacy of fewer than three doses among young women vaccinated against HPV-16 and HPV-18, has shown an efficacy rate of 77% for three-dose schedules, 76% for two-dose schedules, and 85.7% for a single-dose schedule after 4 years of follow-up [89,100,104]. Based on the results, the investigators concluded that the difference in the number of dosages did not result in a significant discordance in the protection against HPV-16 and HPV-18 [89,100,104]. Other studies of Cervarix, Gardasil, Gardasil-9, and Cecolin all found noninferiority with two-dose schedules with the vaccines administered at a 6- or 12-month interval [20].

### 6.3. Single-Dose Schedule

Since many countries worldwide have not yet included the HPV vaccination in their national vaccine programs yet due to financial constraints, an opportunity for a single-dose HPV vaccination is of great interest as it may offer robust protection against vaccine-specific HPV types [10,105].

In the CVT and PATRICIA trial, researchers have demonstrated the efficacy of a single dose of the bivalent HPV vaccine against HPV-16 and HPV-18 [89,100,104]. According to CVT results, protection by a single dose after 7 years of follow-up was sustained with no evidence of diminishing protection [100]. There were no cases of HPV infection during the whole period of follow-up in a single-dose group in comparison to a control group, where the infection rate was 6.6% [100].

Similar results were provided in an Indian trial among 17,729 vaccinated girls, where the immune response against HPV-16 and HPV-18 after a single dose was robust and sustained [98]. However, antibody conversion was significantly lower than in those receiving two and three doses of the vaccine [98]. The aforementioned studies suggest that a single dose of bivalent HPV vaccine may induce sufficient protection [89,90,98,100,104].

The proposal of a single-dose HPV vaccination is included in the HPV-FASTER protocol, which combines HPV screening and vaccination and aims to accelerate the reduction of cervical cancer incidence and mortality [106,107]. According to the HPV-FASTER protocol proposal, the idea is to offer HPV vaccination to females 9–50 years old, irrespective of HPV infection status, along with cervical cancer screening by using any of the approved modalities [106,107,108]. This protocol is suggested for LMICs and has been tested in Mexico [106,108].

However, it is still questionable whether a single-dose or two-dose schedule for older age groups will be sufficient for high and long-lasting immunity [20]. Definitely, a single-dose schedule could have advantages in terms of cost and acceptability; however, it should be investigated.

Currently, the Costa Rica ESCUDDO trial, a double-blind, randomized, noninferiority clinical trial, is running to evaluate and compare the efficacy of one or two doses of the HPV vaccine [90]. The results of the ESCODDO trial are expected to shed more light on the single-dose HPV vaccination efficacy.

## 7. Implementation of Prophylactic HPV Vaccination

Since 2006, when the approval of the first HPV vaccine occurred, 55% of the WHO member states reported implementation of partial or national HPV vaccination [7,17]. According to available data, 40 additional countries have plans to introduce the HPV vaccine in the national schedule by the end of 2023 [17].

Although HPV vaccines are proven to be safe and their efficacy in preventing HPV-related conditions is well recognized [17,63,78,79], the HPV vaccine coverage leaves much to be desired. HPV vaccine safety, adverse reaction concerns, and vaccine hesitancy interfere with the HPV vaccine implementation campaigns worldwide [2].

Many countries have succeeded with national HPV vaccination. There are several high-income countries that developed and implemented strong and sustainable HPV vaccination programs: Australia, the UK (England and Scotland), the USA, New Zealand, Sweden, Denmark, Canada, and Germany [7,109]. An example such as Australia is very encouraging for other countries that are still planning vaccination programs.

Australia was the first country to implement a governmental, population-wide HPV vaccination campaign [72]. The vaccination started in 2007 targeting females aged 12–13 years. In 2007–2009, a catch-up HPV vaccination program was organized using the quadrivalent vaccine Gardasil for all women aged 14–26 years. In 2013, the HPV vaccination program was expanded to males who became similarly eligible for routine vaccination at the target age of 12–13 years [72,110], with an opportunity for a catch-up vaccination program for males aged 14–15 years [72,110]. In January 2018, a two-dose course of the nonavalent HPV vaccine Gardasil-9 was implemented [110]. Subsequently, after the HPV immunization, between 2007 and 2011, the incidence of genital warts declined substantially across Australia, with more than a 90% decline among young women aged < 21 years [72,109]. In addition to the reduction in the genital warts rate in young, vaccinated women, an 82% decline in genital warts among heterosexual males was reported [72,111].

A meta-analysis of the studies from nine developed high-income countries (Australia, Canada, Denmark, England, Germany, New Zealand, Scotland, Sweden, and the USA), with the aim to investigate the population-level impact and herd effects following HPV vaccination, reported a significant decrease in HPV-16 and HPV-18 type prevalence in 68% of countries with HPV vaccination coverage of at least 50% [112]. Subsequently, rates of anogenital warts were reduced by 61% in girls 13–19 years of age. A significant decrease was also recorded for the prevalence of HPV-31, -33, and -45 in this age group of girls, which suggests cross-protection [112]. Moreover, a substantial reduction in rates of anogenital warts was reported in men younger than 20 years of age and in women between 20 and 39 years of age.

The experience of two South American countries, Brazilian and Argentina, with HPV vaccination shows a good example of middle-income countries that developed a successful plan for the HPV vaccine implementation [1,113,114]. The HPV vaccination in Brazil was approved in 2006 and, initially, was available only in private clinics [1,113]. Later, in March 2014, the three-dose schedule with the quadrivalent HPV vaccine was included in the national immunization calendar targeting girls aged 11–13 years old [1]. The HPV vaccination campaign resulted in a 55% decrease in the incidence of genital warts in women younger than 21 years old [1] and no major adverse events were reported.

In Argentina, the HPV vaccine was approved in 2011 for female immunization as part of the national program on cervical cancer prevention [114]. The target group was 11-year-old girls [114]. In 2013, the vaccination coverage of the eligible target group with the first dose reached 87.9%; with the second dose, 71.6%; and with all three doses, 52.2% [114]. However, disparities were reported in HPV vaccine coverage across the country.

However, there are still many countries worldwide that have not developed and implemented HPV vaccination campaigns yet due to limited funding and poor plans for realization [8,17]. Moreover, even countries with well-established vaccination campaigns experience obstacles. For example, the coverage of HPV vaccination in the USA remains low, despite the considerable efforts by the public health agencies [1]. In particular, in 2012, among adolescents aged 13–16 years, only 33.4% of females and 6.8% of males received the three recommended doses of the HPV vaccine [1].

In France, the HPV vaccination program has also experienced barriers to its implementation. France launched the HPV immunization program in 2007, initially covering 14-year-old girls [115]. Later, the catch-up vaccination for females aged 15–23 years became available [115]. However, in France, as in many other countries where HPV vaccination programs are implemented outside of schools, achieving a high HPV vaccination coverage of the target population is a challenging task [115,116]. In 2017, with the launch of the national strategy on sexual health, the aim to achieve 60% HPV vaccine coverage by 2023 and 80% HPV vaccine coverage by 2030 among adolescent girls was set [115,117]. One of the most recent studies among mothers in France revealed that their HPV vaccine perception was affected by conflicting information surrounding HPV vaccination, “focusing on concerns about what appeared to be a widespread belief that these vaccines are unsafe” [118].

Romania is another example of HPV vaccination failure. The HPV vaccination campaign started in 2008, targeting 10–11-year-old girls [119]. However, HPV immunization coverage was as low as 2.57% of the 110,000 eligible girls in the target group [119,120]. Later, in 2010, the campaign was relaunched, targeting 12–14-year-old girls and with a catch-up vaccination available for 16–26-year-old women [119,121]; however, the vaccine coverage remained low (9%) [120]. Parents in Romania rejected the HPV vaccine and the national HPV immunization program was canceled [119]. The program was reinitiated in January 2020 after an 11-year break [120], and the results are yet to be assessed.

Similar issues related to unsuccessful HPV immunization and inappropriate information campaigns on HPV vaccination have occurred in many other countries. The initial Japanese HPV vaccination experience appeared with several reports on side effects, and in June 2013, the Japanese Ministry of Health partially suspended the HPV vaccination campaign [1,122]. A similar situation occurred in Kazakhstan, where HPV vaccination failed in 2013 due to the adverse outcomes negatively and subjectively covered on social media [8]. The French, Japanese, Romanian, and Kazakhstani experiences show that immunization programs can be seriously compromised by adverse social media coverage that is followed by possible “political concerns” [1,8,9,122]. Thus, context-specific health educational interventions sharing accurate information on HPV vaccination safety, effectiveness, and benefits are important to build positive attitudes toward HPV vaccination among parents as decision-makers and should precede a vaccination program [8,123].

## 8. Myths about HPV Vaccination

As was discussed previously, HPV vaccine coverage leave much to be desired in many countries worldwide [2]. There are numerous reasons why parents may reject the HPV vaccination offered to their children. Moreover, even healthcare providers may be hesitant to suggest HPV vaccination [124]. HPV vaccine hesitancy and low acceptance in the majority of cases are attributed to myths and the spread of fake information [113,124,125,126]. These myths and fake information should be dispelled and addressed properly by providing sufficient education based on reliable data [125,126].

Recent publications have identified and summarized several of the most spread myths about HPV vaccination [124,126]. These myths are related to cervical cancer screening, HPV vaccine safety, adverse effects, and the vaccines’ link to infertility, ovarian failure, and autoimmune condition development [126]. Many myths support the idea of vaccines being unnecessarily used for boys/men due to the natural immune system response that clears the infection [124,126]. Moreover, many parents believe vaccination against HPV, as the most common sexually transmitted infection (STI), increases the risk of promiscuity and risky sexual behavior [126]. These fake data disseminated via social media networks already had a huge impact on the vaccination uptake and success of the local HPV vaccination programs [8,113,119].

Dispelling myths and fake information, and providing evidence-based facts related to the HPV vaccination, will help to improve HPV immunization coverage by supporting parents who are willing to provide the best protection for their children [126].

## 9. Challenges and Further Steps in Increasing Preventative HPV Vaccination Coverage

In 2018, the WHO announced the Global Strategy for Cervical Cancer Elimination, which assumes that 90% of girls will be fully vaccinated with any of the available HPV vaccines by the age of 15 [127,128]. Despite significant progress, HPV vaccination coverage has not increased in recent years; moreover, it has dropped since 2020. The coronavirus disease 2019 (COVID-19) pandemic and associated disruptions over the period of 2019–2022 have jolted healthcare systems worldwide, with 25 million children missing out on vaccination in 2021, which is 30% more than in 2019 and the highest number since 2009 [80,127].

Even with the many advantages that HPV immunization brings with its proper implementation, there are many drawbacks and limitations which might impact the vaccination coverage, and thus, should be explained and discussed. These limitations include: (1) the vaccines’ type specificity associated with L1 capsid proteins, specific for each HPV type; (2) the intramuscular or subcutaneous route of administration; (3) the target age group of early adolescence, which creates parental hesitance; (4) the considerable complexity of the vaccines’ formulation and maintenance due to cold-chain storage requirements; (5) the potential communication challenges due to the nature of the HPV infection being an STI and the diversity of cultural/religious beliefs; (6) the cost; and (7) the original three-dose schedule [54,109,126,129,130,131]. The abovementioned factors create economical and healthcare issues for HPV vaccination program implementation, which especially impact LMICs [129].

Each of the drawbacks should be addressed separately to improve the HPV vaccination uptake. The studies on the development of L2 protein-based HPV vaccines may potentially help to overcome HPV vaccines’ type specificity [54,129,130,132]. The high cost of available HPV vaccines, the necessity of their cold-chain maintenance, and the injectable nature of the HPV vaccines could be resolved by the development of dry powder aerosol formulations [131], which will help to reduce economical and healthcare system burdens, especially for LMICs. Availability of an aerosol formulation might help to overcome the potential hesitancy around injection-based vaccination related to cultural and religious beliefs.

HPV vaccine information communication issues and the subsequent low vaccine uptake should be addressed via proper community education information campaigns by addressing parental concerns prior to and/or during the implementation of HPV vaccination, highlighting the vaccines’ safety and effectiveness in girls and boys in preventing HPV-related diseases; this is of paramount importance [17,126,133]. There is a need to continue the dissemination of clear and evidence-based data to further improve vaccination coverage, which will ensure a reduction in HPV-related preinvasive and invasive conditions [109,124,126].

## 10. Conclusions

In 2018, the WHO announced and launched the Global Strategy for Cervical Cancer Elimination, with HPV vaccination being one of the important parts of this campaign. HPV vaccination effectively prevents HPV infection dissemination and the development of associated conditions, which include precancerous cervical lesions, genital warts, and laryngeal papillomas. Although research data show HPV vaccine safety, misinformation leading to HPV vaccination-related concerns has slowed the vaccines’ implementation and coverage in many countries. Moreover, the majority of LMICs do not have enough resources to launch HPV vaccination campaigns. Appropriate educational interventions, adequate implementation plans, and a continuous supply of HPV vaccines could contribute to and facilitate HPV vaccination campaigns and improve the coverage rates.

## Figures and Tables

**Table 1:** Human papillomavirus vaccines’ general characteristics.

Vaccine Type	HPV Types/L1 Included	Brand Name	Producer	Eligible Group	Indications	Ref
Bivalent	HPV-16; HPV-18	Cervarix	GSK, Rixensart, Belgium	Girls and women 9–25 years	Prevention of cervical cancer, CIN grade I-III and AIS, caused by HPV types 16 and 18.	[62]
		Cecolin	Xiamen Innovax Biotech Co., Ltd., Xiamen, China	Girls and women 9–45 years	Prevention of cervical cancer, CIN grade I-III and AIS, caused by HPV types 16 and 18.	[69]
Quadrivalent	HPV-6; HPV-11; HPV-16; HPV-18	Gardasil	Merck Co. Inc., Rahway, NJ, USA	Girls and women 9–26 years	Prevention of: 1. Vulvar and vaginal cancer; 2. Cervical cancer; 3. Genital warts; 4. CIN grade I-III; 5. Cervical AIS; 6. VIN grade II-III; 7. VaIN grade II-III; 8. AIN grades I-III.	[73]
				Boys and men 9–26 years	Prevention of: 1. Genital warts caused by HPV types 6 and 11; 2. Anal cancer and associated precancerous lesions linked to HPV types 6, 11, 16, and 18.	[73]
Nonavalent	HPV-6; HPV-11; HPV-16; HPV-18; HPV-31; HPV-33; HPV-45; HPV-52; HPV-58	Gardasil-9	Merck Sharp & Dohme Corp., Whitehouse Station, NJ, USA	Girls and women 9–45 years	Prevention of: 1.Cervical, vulvar, vaginal, anal, oropharyngeal, and other head and neck cancers, which are caused by HPV types 16, 18, 31, 33, 45, 52, and 58; 2.Genital warts caused by HPV types 6 and 11; 3.CIN grade I-III; 4.Cervical AIS; 5.VIN grade II-III; 6.VaIN grade II-III; 7.AIN grades I-III.	[60]
				Boys and men 9–45 years	Prevention of: 1. Anal, oropharyngeal, and other head and neck cancers caused by HPV types 16, 18, 31, 33, 45, 52, and 58; 2. Genital warts caused by HPV types 6 and 11; 3. AIN grades I-III.	[60]

Abbreviations: HPV—human papillomavirus; CIN—cervical intraepithelial neoplasia; AIS—adenocarcinoma in situ; VaIN—vaginal intraepithelial neoplasia; VIN—vulvar intraepithelial neoplasia; AIN—anal intraepithelial neoplasia.
